# Flood pulse effects on multispecies fishery yields in the Lower Amazon

**DOI:** 10.1098/rsos.150299

**Published:** 2015-11-11

**Authors:** Leandro Castello, Victoria J. Isaac, Ram Thapa

**Affiliations:** 1 Department of Fish and Wildlife Conservation, Virginia Polytechnic Institute and State University, Blacksburg, VA, USA; 2 Department of Forest Resources and Environmental Conservation, Virginia Polytechnic Institute and State University, Blacksburg, VA, USA; 3 Centro de Ciências Biológicas, Universidade Federal do Pará, Belém, Pará, Brazil

**Keywords:** feeding strategies, modelling, population dynamics, seasonal inundation, tropics

## Abstract

Seasonally fluctuating water levels, known as ‘flood pulses’, control the productivity of large river fisheries, but the extent and mechanisms through which flood pulses affect fishery yields are poorly understood. To quantify and better understand flood pulse effects on fishery yields, this study applied regression techniques to a hydrological and fishery record (years 1993–2004) for 42 species of the Amazon River floodplains. Models based on indices of fishing effort, high waters and low waters explained most of the interannual variability in yields (*R*^2^=0.8). The results indicated that high and low waters in any given year affected fishery yields two and three years later through changes in fish biomass available for harvesting, contributing 18% of the explained variability in yields. Fishing effort appeared to amplify high and low water effects by changing in direct proportion to changes in fish biomass available for harvesting, contributing 62% of the explained variability in yields. Although high waters are generally expected to have greater relative influence on fishery yields than low waters, high and low waters exerted equal forcing on these Amazonian river-floodplain fishery yields. These findings highlight the complex dynamics of river-floodplain fisheries in relation to interannual variability in flood pulses.

## 1. Introduction

Seasonally fluctuating water levels, known as ‘flood pulses’, control the structure and function of large river ecosystems [1]. Flood pulses promote high rates of biological production and drive the generally high productivity of large river fisheries that provide food and income to millions of people globally [2–4]. Yet, the extent and mechanisms through which flood pulses affect fishery yields are poorly understood, impeding assessment and prediction of the impacts on fisheries caused by river hydrological alterations (e.g. by dams).

Previous studies have shown that flood pulses influence the dynamics of fish populations and associated fishery yields. Rising water levels trigger fish production processes, as many fish species spawn and migrate laterally out of river channels onto the newly flooded floodplains when water levels rise [5,6]. In the floodplains, fish growth and recruitment rates generally increase as fish find protection from predators and abundant plant-based food resources, including algae, detritus, and tree fruits and seeds [7,8]. Conversely, declining water levels trigger mortality processes by constraining fish to river channels and floodplain lakes, where increased fish densities intensify predation rates and water quality is often poor [4,9–14]. Interannual variability in flood pulses thus influences fishery yields. Previous studies have found that extreme high water years can increase biomass for harvesting in subsequent years by promoting fish recruitment and growth rates; conversely, extreme low water years can reduce biomass for subsequent harvesting by increasing natural mortality rates [13,15,16]. Flood pulse indices in a given year have been correlated with annual multispecies yields or standing biomass in subsequent years: 92% in the Niger, 82% in the Shire, 57% in the Kafue and 83% in the Amazon [4,17].

A dearth of studies on the topic has left unanswered several questions about the role of fishing effort, life-history traits, and high and low waters in influencing fishery yields. This is key as fisheries in river floodplains with near-pristine hydrological cycles exhibit large interannual variation in yields (e.g. up to 400% [13]). First, the extent to which these fishery yields are governed by interannual variability in flood pulses in addition to fishing effort is unknown. Correlations of flood pulse indices and fishery yields alone, as done in previous studies, cannot explain variation in annual multispecies yields, because both fishing effort and yields must be considered as each typically varies among and within years. The large yields observed in, or following, extreme high water years, for example, could be due to fishing effort being greater than normal.

Second, previous studies have focused on the species with short lifespans and high fecundities (e.g. *Puntius sophore*) that dominate river-floodplain fisheries in Africa [16], leaving unknown flood pulse affects on fishery yields of species with different life-history traits. Various life-history traits are found in species dominating fishery yields in many river floodplains (e.g. South America [18]). A life-history trait that may explain fish population responses to flood pulse variability is feeding strategy, because it defines energy sources for the individuals and it has been used to predict anthropogenic effects on stream fishes [19].

Finally, most studies have reported positive correlations between high waters in one year and fishery yields in subsequent years, leading to the prevailing notion that ‘fish yields and production are strongly related to the extent of accessible floodplain’ [1]. This suggests that river-floodplain fishery yields are controlled by high waters, which are associated with production processes, and not by low waters, which are associated with mortality processes. This notion is consistent with findings that fishery yields in marine and freshwater ecosystems are mainly controlled by primary productivity [20,21]. However, the relative strength of the influence of high and low waters on river-floodplain fishery yields has not been assessed.

This study addressed the following questions: (i) do flood pulses influence multispecies fishery yields when fishing effort is accounted for? (ii) Are fishery yield responses to flood pulse variability explained by feeding strategies? (iii) What is the relative strength of the influence of high and low waters on fishery yields? These questions were addressed through analyses of 12 years of data on water levels and fishery yields and effort for 42 species in a river-floodplain of the Amazon Basin that is near-pristine hydrologically. The analyses were based on regression models that included indices of effort and low and high waters as candidate explanatory variables and yields of all species, piscivores, detritivores, omnivores or herbivores as the response.

## 2. Methods

**Figure 1.**
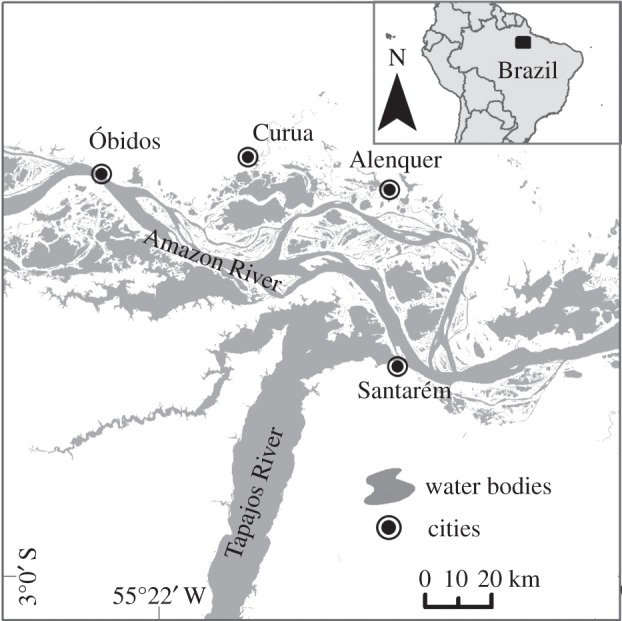
River floodplains of the Lower Amazon mainstem near the municipalities of Santarém, Óbidos and Alenquer, where fisheries landing data were collected.

The study area is the Lower Amazon mainstem in the State of Pará, Brazil (figure 1). Here, mean flood pulse amplitude is 7 m with a maximum water level in June and a minimum in October; the floodplains are classified as *várzea*, a type of floodplain that flanks the sediment-rich whitewater rivers of the Amazon [22]. Fishing occurs year-round, but fishery yields are highest between March and October [23].

### 2.1 Data sources

Fishery yields and effort data were collected daily between January 1993 and December 2004 over an approximately 250 km stretch of river. Only data from 54 798 fishing trips involving motorized boats and gillnets were used, to reduce additional variance caused by different catchability rates among capture methods (electronic supplementary material, table S1). Motorized boats and gillnets contributed 52% of recorded fishery yields.

**Figure 2.**
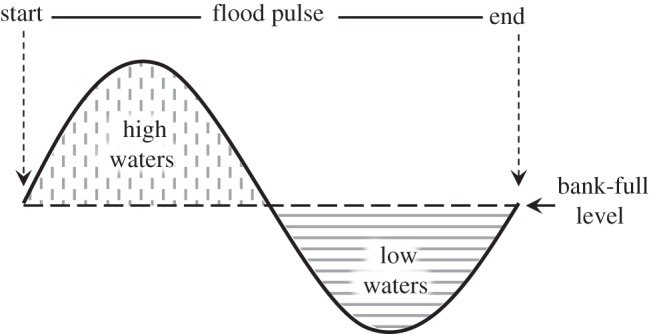
Schematic diagram of the flood pulse and areas of the hydrograph curve used to calculate high (*H*) and low (*L*) water indices. *H* and *L* were calculated using daily river water levels at Óbidos city (figure 1) for the period between 1990 and 2004.

**Figure 3.**
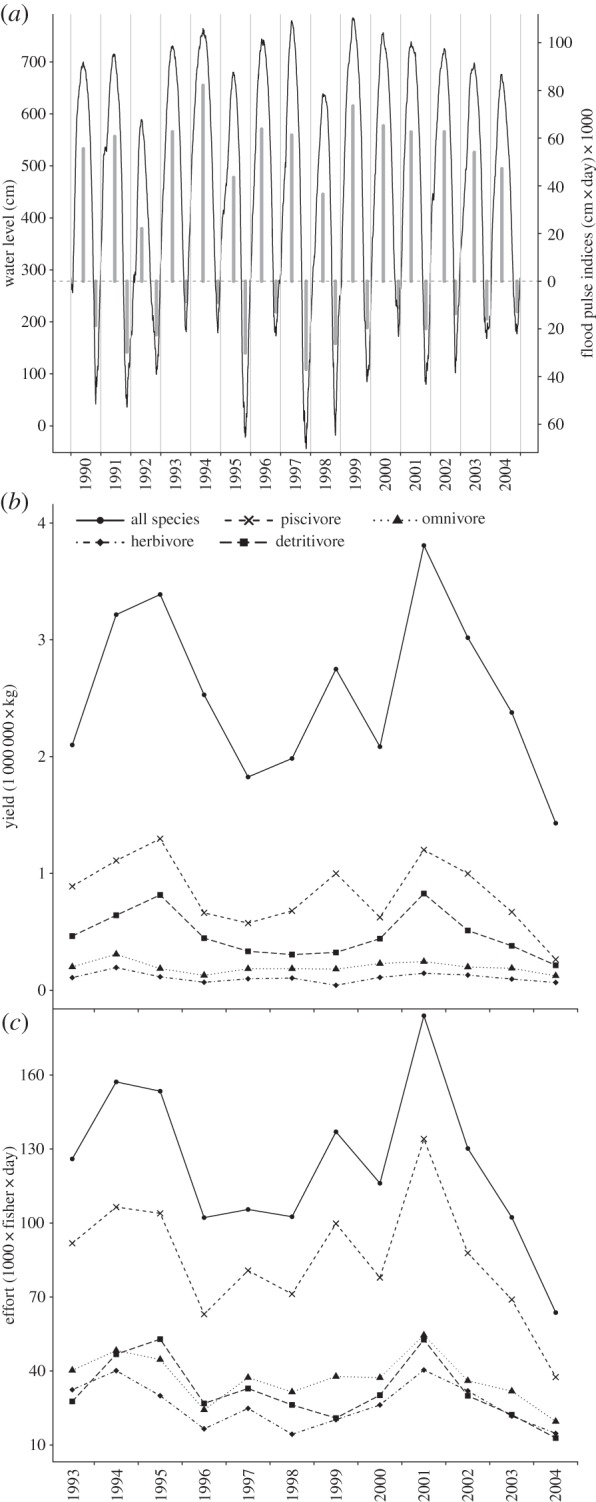
(*a*) Daily water levels (black line) and respective high (*H*) and low (*L*; grey bars) water indices during the study period. (*b*) Fishery yields and (*c*) respective fishing effort during the study period.

Species yields were grouped into four feeding strategies (piscivores, omnivores, detritivores and herbivores) based on literature data ([24,25]; electronic supplementary material, table S2). High and low water indices, denoted by *H* and *L*, respectively, were calculated as the area under and above the hydrograph curve, respectively, relative to a ‘bank-full’ level, based on Welcomme [4] (figure 2). The bank-full is the level at which rising waters, on average, overflow river channels and flood the floodplains; a first estimate of bank-full for the study area was estimated by linking field estimates of flooding of vegetated floodplain habitats to historical water level data. Habitats of low swampy woodland (i.e. *chavascal*) flooded at 0.42 and 0.45, respectively, of water-level differences between minimum and maximum during the years the studies were conducted [6,26]. Bank-full was estimated applying the average of these flooding values to the difference between minimum and maximum water levels during the study period for the Obidos data (figure 3 and electronic supplementary material, table S3).

### 2.2 Data analyses

The effects of *H*, *L*, fishing effort, and feeding strategies on fishery yields were investigated using linear regression models. Model variables included annual yield as the response and annual fishing effort and six flood pulse indices as candidate explanatory variables. The flood pulse indices included were *H*_−1_, *H*_−2_, *H*_−3_ and *L*_−1_, *L*_−2_, *L*_−3_, denoting *H* and *L* in one, two or three years, respectively, before the year in which fishing took place. Such time lags can account for hydrological effects on fish age zero or older because they encompass the age range of the specimens dominating yields, which is around 2 years [18]. This age at capture is due to generally fast growth rates and moderate exploitation rates [18,27]. Flood pulse indices in the year in which fishing took place (e.g. *H*_0_) were not included to avoid possible confounding effects created by water level seasonality on catchability rates [4]. Years with intense high or low waters affect catchability rates through changes in habitat area and overall fish densities, having an effect on catch regardless of potential effects of high and low waters in prior years on fish biomass available for harvesting. By not considering flood pulse indices in the year in which fishing took place, the model structure focused on flood pulse effects on increasing or decreasing fish biomass available for harvesting. In doing this, it was assumed that interannual variation in catchability rates is on average null, an assumption that probably holds given the presence of intense high and low water years during the study period (figure 3). Candidate models were constrained to have a maximum of four explanatory variables and all the main effects were included in the model when their interaction was significant (e.g. effort in a given year may change in response to *H* or *L* the previous year).

Annual yields were not serially autocorrelated (Box–Pierce test at *p*=0.1). Except for a mild correlation between effort and *L*_−1_, there were no correlations among candidate variables, including effort in the year in which fishing took place (which was considered in the models) and effort one and two years prior (which were not considered in the models; electronic supplementary material, table S4). Yields in the year in which fishing took place were not correlated to effort in prior years (*p*>0.05), indicating that yields were not influenced by fishing mortality in previous years. *H* and *L* indices were not correlated, because annual and seasonal river discharge varied interannually.

Model selection was based on the information-theoretic approach of Burham & Anderson [28], which allows evaluation of evidence in observational data for multiple working hypotheses. Each candidate model was considered a working hypothesis. Analyses were performed in R v. 3.2.0, including packages ‘glmulti’ [29] and ‘MuMIn’ [30] for model averaging. Akaike’s information criterion corrected (AICc) for small samples was used to select best approximating models. Akaike weights (*w*_*i*_), defined as the weight of evidence in favour of model *i* being the actual best model for a set of models, were calculated for all models. *w*_*i*_ is calculated as

$$w_{i}=\frac{\exp(-1/2\Delta_{i})}{\sum_{n=1}^{r}\exp(-1/2\Delta_{n})},$$

where *r* is the number of best approximating models in the candidate set, and Δ_*i*_=AICc_*i*_−AICc_min_. AICc_min_ is the lowest AICc value among *r* models. *w*_*i*_ values range from zero to one, and the sum of weights of all competing models in the candidate set is one. Models were ranked based on *w*_*i*_. Only models possessing Δ values less than two were considered to have substantial support from the data. When there was more than one ‘best’ approximating model, model parameter estimates were obtained from model averaging based on all best approximating models. Model-averaged parameter estimates with confidence intervals that included zero were assumed to have little support. Best approximating models were assessed with respect to multicollinearity (variance inflation factor), independent errors (Durbin–Watson test), normally distributed errors (Shapiro–Wilk test and visual inspection of residual plots), and influential cases (Cook’s distance). Appropriate transformations were applied to yields and effort data when there was error non-normality or non-constant variance. Effect sizes were calculated based on semi-partial *R*^2^ for all explanatory variables. All analyses used type I error *α*=5%.

To assess if flood pulses influence multispecies fishery yields when fishing effort is accounted for, model selection was applied to an initial candidate model including data for all species (referred to as ‘all-species’), respective fishing effort, and *H* and *L* indices. To assess if feeding strategies explain fish population responses to flood pulse variability, candidate models were as above with the difference that the responses were yields of piscivores, omnivores, detritivores and herbivores. Finally, to quantify the relative strength of the influence of high and low waters on fishery yields, the percentage change in yields caused by a 100% change in mean *H* and *L* values, done one at a time, was estimated at mean values of all other explanatory variables. These predictions were based on model-averaged parameter estimates from models within a 95% confidence set, which is sum of *w*_*i*_ from the largest weight until the sum is 0.95. Mann–Whitney *U*-tests compared the % change in yields to 100% changes in *H* and *L* values.

## 3. Results

### 3.1 Model diagnostics

Log-10 transformations were applied to yields and effort datasets of the all-species and per guild fish groups, except to yields of detritivores in which a power (−1) transformation was required. Data for the planctivore *Hypophthalmus* spp. were maintained in the all-species model, but excluded from per feeding strategy models because their residuals were highly nonlinear and heterocedastic, regardless of data transformations. Variance inflation factor values were less than four, and Durbin–Watson statistics was close to two for all models. The Shapiro–Wilk test indicated error non-normality in the second best model for omnivores and second and fourth best models for herbivores (*p*<0.01). Visual inspection of residual plots showed no patterns (electronic supplementary material, figure S1). Cook’s distance plots indicated that an observation in 1999 in the third best model for herbivores could be influential, but that observation was maintained because it represented an extreme high water year (figure 3).

### 3.2 Modelling analysis

Yield and effort varied 370% and 324%, respectively, on average across all fish groups, whereas *H* and *L* varied 373% and 433%, respectively; mean coefficients of variation were 28% for *H*, 46% for *L*, 34% for yields and 32% for effort (electronic supplementary material, tables S5 and S6). Effort, *H* and *L* explained most of the interannual variability in yields in the selected models (mean *R*^2^=0.8; table 1). *H* and *L* contributed on average 18%, and effort 62%, of the explained variability in yields across all fish groups (electronic supplementary material, table S7).

**Table 1.** Best approximating models for each fish group based on AICc values, AICc differences from best model (Δ) and Akaike’s weights (*w*_*i*_). *K* is number of parameters estimated for each model, including intercept and error terms.

fish group	models	*R*^2^	log-likelihood	AICc	Δ	*w*_*i*_	*K*
all-species	effort, *L*_−2_	0.91	22.64	−31.57	0.00	1.00	4
piscivores	effort, *L*_−2_	0.92	18.93	−24.15	0.00	0.62	4
	effort	0.88	16.08	−23.16	0.99	0.38	3
omnivores	effort, *H*_−2_	0.88	22.98	−32.25	0.00	0.56	4
	effort, *L*_−3_, *H*_−3_	0.93	25.90	−31.79	0.46	0.44	5
herbivores	effort, *L*_−2_, *H*_−2_	0.81	14.63	−9.26	0.00	0.25	5
	effort	0.53	9.09	−9.18	0.08	0.24	3
	effort, *L*_−2_	0.68	11.41	−9.10	0.16	0.23	4
	effort, *L*_−3_	0.66	11.03	−8.34	0.92	0.16	4
	effort, *H*_−1_	0.65	10.86	−8.00	1.26	0.13	4
detritivores	effort	0.82	159.75	−310.50	0.00	1.00	3

**Figure 4.**
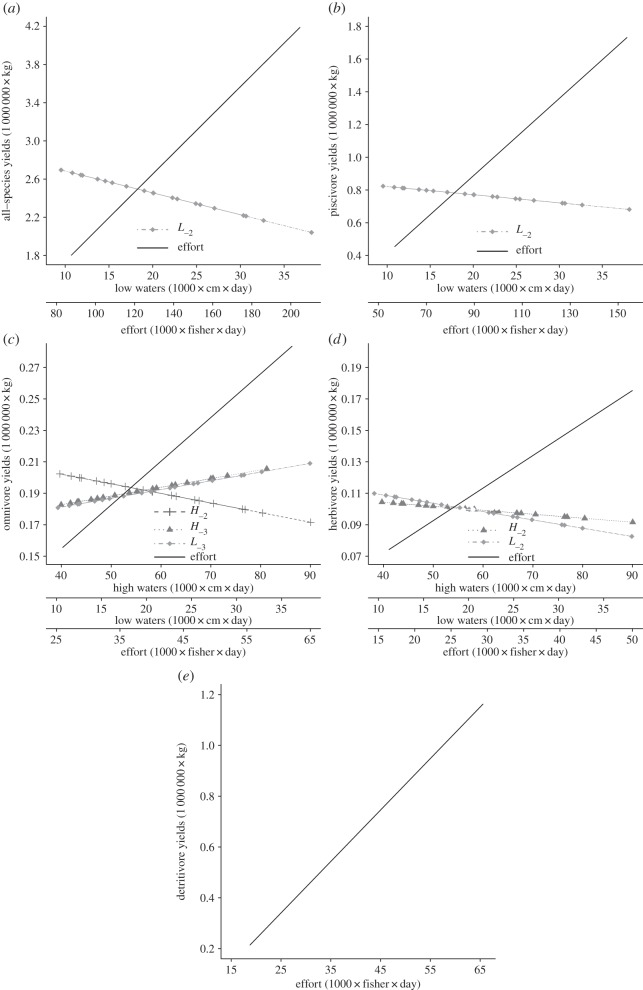
*H*, *L* and effort effects on yields of (*a*) all-species, (*b*) piscivores, (*c*) omnivores, (*d*) herbivores and (*e*) detritivores. Plots derived from the best approximating or full-average models presented in table 2.

There was only one best approximating model for all-species (table 1), which indicated that effort had a positive effect and *L*_−2_ had a negative effect on yields (table 2 and figure 4).

**Table 2.** Parameter estimates and associated confidence intervals (in parentheses) of best approximating or averaged models. Parameter estimates and confidence intervals were computed using regular regression method when there was only one model within a Δ of two, and using a model averaging procedure when there was more than one model. Parameters marked by asterisk had little support following calculation of unconditional confidence intervals. All *H* and *L* parameters represented at 10^−6^ unless noted otherwise.

fish group	intercept	effort	*L*_−1_	*H*_−1_	*L*_−2_	*H*_−2_	*L*_−3_	*H*_−3_
all-species	1.43	0.99			−4.15			
	(0.19, 2.66)	(0.74, 1.23)			(−7.29, −0.99)			
piscivores	−0.53	1.32			−4.58			
	(−2.07, 1.00)	(0.99, 1.63)			(−9.01, −0.15)			
omnivores	2.12	0.69				−2.53	5.10	2.31
	(0.78, 3.46)	(0.44, 0.94)				(−4.35, −0.70)	(2.11, 8.09)	(0.63, 3.98)
herbivores	1.68	0.78		3.72*	−8.97	−4.53	7.13*	
	(−0.55, 3.92)	(0.27, 1.28)		(−1.07, 8.50)	(−17.24, −0.70)	(−8.90, −0.15)	(−1.58, 15.83)	
detritivores^a^	2.50	−0.50						
	(1.75, 3.24)	(−0.67, −0.34)						

^a^Model parameters based on power (−1) transformation of yield data; parameter estimates and standard errors represented at 10^−5^ for intercept and effort.

The best model for piscivores included effort and *L*_−2_, and the second best model included only effort (table 1). Model-averaged parameter estimates for piscivores indicated that effort had a positive effect and *L*_−2_ had a negative effect on yields (table 2 and figure 4).

The best model for omnivores included effort and *H*_−2_, and the second best model included effort, *H*_−3_ and *L*_−3_ (table 1). Model-averaged parameter estimates for omnivores indicated that effort had a positive effect, *H*_−2_ had a negative effect and *H*_−3_ and *L*_−3_ had positive effects on yields (table 2 and figure 4).

The best model for herbivores included effort, *L*_−2_ and *H*_−2_; the second best model included only effort; the third included effort and *L*_−2_; the fourth included effort and *L*_−3_; and the fifth included effort and *H*_−1_ (table 1). Model-averaged parameter estimates indicated there was little support for the estimates of *H*_−1_ and *L*_−3_, and that effort had a positive effect and *L*_−2_ and *H*_−2_ had negative effects on yields (table 1 and figure 4).

The best model for detritivores included only effort (table 1). Because yields of detritivores were transformed to the power of −1, parameter estimates indicated effort had a positive effect on yields (table 2 and figure 4).

### 3.3 Sensitivity analysis

Changes in yields caused by a 100% change in mean *H* and *L* were no different (Mann–Whitney *U*-test, *p*>0.05) across all fish groups, feeding strategy groups and all individual indices (table 3).

**Table 3.** Sensitivity analysis of a 100% change in flood pulse indices on predicted yields. The response effect in yields (measured in %) was calculated with respect to the range of response observations (i.e. max.–min.) to facilitate interpretation. Calculations done on model-averaged parameter estimates within a 95% confidence set. Some *H* and *L* indices included in this analysis were not included in table 2, because those average models were calculated based on all best approximating models within an AICc interval of two. Median values calculated based on absolute values of response effect.

fish group	*H*_−1_	*L*_−1_	*H*_−2_	*L*_−2_	*H*_−3_	*L*_−3_
all-species	1.2	-0.1	4.1	-15.6	0.4	-1.8
piscivores	-1.1	0.3	2.5	-7.4	-0.8	-0.2
omnivores	0.1	0.1	-20.2	-0.1	-11.1	10.4
herbivores	7.5	-0.5	-7.8	-11.3	-0.4	4.4
detritivores	2.5	-0.1	-1.7	0.1	-0.2	1.0
median	1.15	0.11	4.09	7.40	0.44	1.82

## 4. Discussion

These results contribute to understanding the complex interannual dynamics of river-floodplain fish populations and fisheries. Four of the five models of fishery yields included effort and flood pulse indices, indicating that flood pulses affect multispecies fishery yields even when fishing effort is considered. *H* and *L* in any given year affected fishery yields two and three years later, presumably via increases and decreases in fish biomass available for harvesting, contributing 18% of the explained variability in yields. Although effort was not statistically related to *H* and *L*, changes in fish biomass driven by *H* and *L* appear to have caused effort to vary in a direct manner, contributing 62% of the explained variability in yields. Years with large amounts of fish biomass attracted high levels of fishing effort, and years with small amounts of fish biomass attracted low levels of fishing effort. Therefore, both high and low waters affect biomass available for harvesting, and fishing effort appears to respond to such biomass changes by amplifying their effects on fishery yields.

Fishery yield responses to flood pulse variability varied by feeding strategy. As expected based on previous studies, *L*_−2_ had negative effects on biomass available for harvesting in yield models of all-species, piscivores and herbivores, and *H*_−3_ had a positive effect on fish biomass in the yield model of omnivores. However, *L*_−3_ had a positive effect on fish biomass in the yield model of omnivores, and *H*_−2_ had negative effects on fish biomass in the yield models of omnivores and herbivores, indicating that low waters do not always decrease, and high waters do not always increase, fish biomass. There was no evidence of flood pulse effects on yields of detritivores.

The negative effects of *L*_−2_ on yields of all-species, piscivores and herbivores indicate that low waters significantly reduce fish biomass available for harvesting by promoting natural mortality. Most species in these groups inhabit floodplain lakes permanently (e.g. *Plagioscion squamosissimus*) or temporarily (e.g. *Mylossoma duriventre*), including the migratory catfishes (e.g. *Brachyplatystoma rousseauxii*) that dominated yields of piscivores and whose adults mainly inhabit river channels [7,31]. Mortality rates are often high in floodplain lakes during low waters. Habitat reductions intensify predation rates, and, combined with high temperatures, they usually decrease pH and dissolved oxygen levels and increase nutrient content, causing fishes to enter torpor or die [9–14,32]. This occurs even in the tropics where fish are physiologically adapted to tolerate poor water quality [33]. Migratory catfishes can also be adversely affected by low waters through decreased prey availability. The observed negative effect of *L*_−2_ on yields is consistent with another study that found multispecies fish biomass (i.e. capture per unit effort) in a given year to be negatively linked to low waters two years prior in river floodplains in the Central Amazon [17]. The importance of low water mortality for tropical floodplain fish assemblages was shown by another study in the Central Amazon. Out of 14 environmental variables, floodplain lake depth was found to be the strongest predictor of the abundance of *Arapaima* spp., during low waters, as the fish selected the deepest lakes to maximize survival rates in drought years [34].

The negative effects of *H*_−2_ on yields of herbivores and omnivores are probably owing to the low oxygen conditions often found in floodplain habitats during high waters. The dominant fishes in these two feeding strategies (*Metynnis*spp., *Astronotus crassipinnis*, *Colossoma macropomum* and *Schizodon fasciatus*) were mostly larvae early in *H*_−2_ [35,36]. Dissolved oxygen can be very low in the floodplains during high waters owing to stratification of the water column [12]. Low oxygen levels could limit access of the young to feeding grounds or lower their survival rates.

The positive effects of *L*_−3_ and *H*_−3_ on yields of omnivores indicate recruitment controls. The two taxa dominating yields of omnivores feed more intensely at different times of the flood cycle. *C. macropomum* feeds more actively during high waters [7], whereas *S. fasciatus* feeds more actively during low waters [37]. Therefore, *H*_−3_ could regulate feeding opportunities for adult individuals of *C. macropomum*, thereby influencing their reproductive output; and *L*_−3_ could regulate feeding opportunities for adult individuals of *S. fasciatus*, similarly influencing their reproductive output. The effect of *H*_−3_ on yields of omnivores is consistent with the finding from a previous study in river floodplains of the Central Amazon that multispecies fish biomass in a given year is positively linked to high waters three years prior [17]. The lack of an effect of *L*_−3_ in that study in the Central Amazon could be due to the prevalence of *C. macropomum* in fishery yields relative to that played by *C. macropomum* in this study, where yields of *S. fasciatus* and other related taxa were significant [36].

Flood pulse effects on yields of detritivores were expected given that this group is dominated by ‘seasonal strategists’ species, such as *Prochilodus nigricans*. Seasonal strategist species exhibit large clutches and small investment per offspring, so their populations generally expand and shrink quickly in response to habitat conditions [38]. The lack of flood pulse effects on yields of detritivores could be due to the existence of multiple age cohorts confounding *H* and *L* effects. It also could be due to the presence in yields of species with different migratory and reproductive strategies, including the siluriform *Pterygoplichthys pardalis* and the characiforms *Prochilodus nigricans* and *Semaprochilodus* sp.

High waters were expected to dominate flood pulse effects on fishery yields, but high and low waters exerted equal forcing on Amazonian multispecies river-floodplain fishery yields. The effects of low waters on river-floodplain fishery yields documented here indicate they are seasonal phenomena that produce impacts on fish populations analogous to those produced by extreme drought events in streams on an interannual or even interdecadal basis [9]. In line with this finding is a study in the Kafue River in Africa showing that low waters of an average hydrological year decreased by 40% the fish biomass found in the preceding high waters [12].

Many studies have explained the impacts of river hydrological alterations on river-floodplain fisheries solely based on habitat degradation and blocked longitudinal migrations, paying little attention to flood pulse effects on fish population dynamics. Mining of water aquifers, water diversion, wetland drainage, desertification, deforestation and dam building all have altered river hydrology worldwide mainly by decreasing the magnitude of high waters and increasing the magnitude of low waters [39]. The results herein indicate that such hydrological alterations impact fish populations, not only via degraded habitat and blocked longitudinal migrations, but also largely via intensification of natural mortality processes and weakening of body growth and recruitment processes. This study shows that the droughts that are becoming more frequent and intense in many river floodplains owing to escalating hydrological alterations (e.g. the Amazon [40]) affect strongly and adversely the world’s most productive freshwater fisheries.

## Supplementary Material

Supplementary Material.docx

## Data accessibility

The datasets supporting this article are provided in the electronic supplementary material.
