# Supplementary material for: Flood pulse effects on multispecies fishery yields in the Lower Amazon
Source: R Soc Open Sci. 2015 Nov 11;2(11):150299. doi: 10.1098/rsos.150299 (PMC4680609; doi:10.1098/rsos.150299)
Supplement: Supplementary Material.docx [file rsos150299supp1.docx]

# Supplementary Material:

## Flood pulse effects on multispecies fishery yields in the Lower Amazon

**Authors:**

Leandro Castello^1,*^, Victoria J. Isaac^2^, Ram Thapa^3^

**^1^** Department of Fish & Wildlife Conservation, Virginia Polytechnic Institute and State University, Blacksburg, Virginia, USA

**^*^** Corresponding author: [leandro@vt.edu](mailto:leandro@vt.edu), Tel: +1-540-231-5046

**^2^** Centro de Ciências Biológicas, Universidade Federal do Pará, Belém, Pará, Brazil

**^3^** Department of Forest Resources and Environmental Conservation, Virginia Polytechnic Institute and State University, Blacksburg, Virginia, USA

**Appendix figure 1.** Residual plots of the best approximating models presented in Table 1.

**Appendix table 1.** Annual yield and fishing effort data.

| Year | Piscivore | Omnivore | Herbivore | Detritivore | All-species |
| --- | --- | --- | --- | --- | --- |
|  | Yield (kg) | Yield (kg) | Yield (kg) | Yield (kg) | Yield (kg) |
| 1993 | 889,888 | 200,040 | 108,105 | 463,911 | 2,099,911 |
| 1994 | 1,110,024 | 308,966 | 194,309 | 641,853 | 3,215,444 |
| 1995 | 1,296,824 | 185,347 | 115,085 | 815,881 | 3,388,305 |
| 1996 | 662,605 | 128,075 | 68,403 | 446,347 | 2,529,002 |
| 1997 | 574,928 | 183,985 | 98,069 | 333,785 | 1,825,721 |
| 1998 | 679,781 | 184,429 | 104,359 | 305,660 | 1,984,401 |
| 1999 | 999,701 | 180,992 | 42,684 | 323,799 | 2,749,368 |
| 2000 | 624,771 | 229,224 | 110,111 | 441,656 | 2,085,055 |
| 2001 | 1,201,919 | 245,294 | 145,817 | 827,623 | 3,807,848 |
| 2002 | 998,501 | 199,125 | 130,238 | 511,435 | 3,018,078 |
| 2003 | 670,448 | 188,048 | 95,959 | 380,482 | 2,377,550 |
| 2004 | 263,748 | 123,929 | 66,382 | 213,316 | 1,430,352 |
|  | Effort (fishers*days) | Effort (fishers*days) | Effort (fishers*days) | Effort (fishers*days) | Effort (fishers*days) |
| 1993 | 91,791 | 40,291 | 32,421 | 27,655 | 125,998 |
| 1994 | 106,493 | 48,311 | 40,131 | 46,816 | 157,231 |
| 1995 | 103,931 | 44,666 | 29,974 | 52,916 | 153,457 |
| 1996 | 63,037 | 24,230 | 16,565 | 26,871 | 102,198 |
| 1997 | 80,732 | 37,340 | 24,845 | 32,909 | 105,501 |
| 1998 | 71,206 | 31,503 | 14,347 | 26,244 | 102,558 |
| 1999 | 99,758 | 37,796 | 20,277 | 20,922 | 136,994 |
| 2000 | 77,882 | 37,295 | 26,236 | 30,203 | 116,129 |
| 2001 | 134,094 | 54,526 | 40,417 | 52,728 | 184,049 |
| 2002 | 87,835 | 36,042 | 31,953 | 29,957 | 130,228 |
| 2003 | 68,974 | 31,831 | 21,727 | 22,278 | 102,294 |
| 2004 | 37,526 | 19,554 | 14,705 | 12,829 | 63,690 |

**Appendix table 2.** Fish species, feeding strategies, and average yield contribution in weight during the study period.

|  |  |  |  |
| --- | --- | --- | --- |
| Common name | Scientific name | Feeding guild | Yield |
| Mapara | *Hypophthalmus edentatus, H. marginatus* | Planktivore | 35% |
| Dourada | *Brachyplatystoma rousseauxii* | Piscivore | 15% |
| Curimata | *Prochilodus nigricans* | Detritivore | 13% |
| Fura Calça | *Pimelodina flavipinnis* | Piscivore | 8% |
| Pescada | *Plagioscion squamosissimus; P. surinamensis* | Piscivore | 8% |
| Jaraqui | *Semaprochilodus taeniurus, S. insignis* | Detritivore | 8% |
| Acari | *Squaliforma emarginata, Pterygoplichthys pardalis* | Detritivore | 8% |
| Surubim | *Pseudoplatystoma fasciatum, P. tigrinum* | Piscivore | 6% |
| Aracu | *Schizodon fasciatus, S. vittatus, Leporinusspp., Rhytiodus microlepis, Rhytiodus argenteofuscus, Laemolita taeniata* | Omnivore | 5% |
| Tambaqui | *Colossoma macropomum* | Omnivore | 5% |
| Piramutaba | *Brachyplathystoma vaillantii* | Piscivore | 4% |
| Pacu | *Metynnis spp., Mylossoma duriventre, M. aureum* | Herbivore | 4% |
| Filhote | *Brachyplatystoma filamentosum* | Piscivore | 4% |
| Acara | *Astronotus crassipinnis, Geophagus proximus* | Herbivore | 2% |
| Tucunare | *Cichla monoculus, Cichla sp.* | Piscivore | 2% |
| Apapa | *Pellona flavipinnis, P. castelnaeana* | Piscivore | 1% |
| Matrinchã | *Brycon spp.* | Omnivore | 1% |
| Cujuba | *Oxydoras niger* | Piscivore | 1% |
| Pirapitinga | *Piaractus brachypomus* | Omnivore | 1% |
| Barbado | *Brachyplatystoma platynemum* | Piscivore | <1% |
| Aruana | *Osteoglosum bicirrhosum* | Piscivore | <1% |
| Bacu | *Pterodoras granulosus, Lithodoras dorsalis* | Herbivore | <1% |
| Charuto | *Bivibranchia sp, Hemiodus spp., Anodus elongatus* | Herbivore | <1% |
| Pirarara | *Phractocephalus hemioliopterus* | Omnivore | <1% |
| Jau | *Zungaro zungaro* | Piscivore | <1% |
| Pirarucu | *Arapaima spp.* | Piscivore | <1% |
| Branquinha | *Curimata spp., Curimatella spp. Cyphocharax spp., Psectrogaster spp., Potamorhina altamazonica, Potamorhina latior* | Omnivore | <1% |
| Mandube | *Ageneiosus inermis* | Piscivore | <1% |
| Tamoata | *Hoplosternum littorale* | Detritivore | <1% |
| Piranambu | *Pinirampus pirinampu* | Piscivore | <1% |
| Piranha | *Pygocentrus nattereri, Pristobrycon spp., Serrasalmus spp.* | Piscivore | <1% |
| Sardinha | *Triportheus spp.* | Omnivore | <1% |
| Traira | *Hoplias malabaricus* | Piscivore | <1% |
| Piracatinga | *Calophysus macropterus* | Piscivore | <1% |
| Jandia | *Leiarius marmoratus* | Piscivore | <1% |
| Arraia | *Potamotrygon motoro, Potamotrygon sp.* | Piscivore | <1% |
| Peixe-Cachorro | *Hydrolycus scomberoides* | Piscivore | <1% |
| Cara de Gato | *Platynematichthys notatus* | Piscivore | <1% |
| Mandi | *Pimelodus blochii* | Omnivore | <1% |
| Saranha | *Cynodon gibbus, Rhaphiodon vulpinus* | Piscivore | <1% |
| Jeju | *Hoplerythrinus unitaeniatus* | Piscivore | <1% |
| Poraque | *Electrophorus electricus* | Piscivore | <1% |

**Appendix table 3.**  Flood pulse indices used in the analyses.

| Year | *H_-1_* | *L_-1_* | *H_-2_* | *L_-2_* | *H_-3_* | *L_-3_* |
| --- | --- | --- | --- | --- | --- | --- |
| 1993 | 22,035 | 22,438 | 60,665 | 29,680 | 55,527 | 18,552 |
| 1994 | 62,667 | 8,520 | 22,035 | 22,438 | 60,665 | 29,680 |
| 1995 | 82,123 | 8,832 | 62,667 | 8,520 | 22,035 | 22,438 |
| 1996 | 43,503 | 30,081 | 82,123 | 8,832 | 62,667 | 8,520 |
| 1997 | 63,766 | 12,845 | 43,503 | 30,081 | 82,123 | 8,832 |
| 1998 | 61,256 | 36,907 | 63,766 | 12,845 | 43,503 | 30,081 |
| 1999 | 36,435 | 26,084 | 61,256 | 36,907 | 63,766 | 12,845 |
| 2000 | 73,474 | 19,441 | 36,435 | 26,084 | 61,256 | 36,907 |
| 2001 | 65,124 | 11,066 | 73,474 | 19,441 | 36,435 | 26,084 |
| 2002 | 62,554 | 19,834 | 65,124 | 11,066 | 73,474 | 19,441 |
| 2003 | 62,659 | 13,569 | 62,554 | 19,834 | 65,124 | 11,066 |
| 2004 | 54,008 | 15,777 | 62,659 | 13,569 | 62,554 | 19,834 |

**Appendix table 4.** Correlation matrix of effort in the year in which fishing took place (Eff), effort one and two years prior (Eff_-1_, Eff_-2_, respectively), and high and low water indices one, two and three years prior (*H_-1_, H_-2_, H_-3_, L_-1_, L_-2_, L_-3_*, respectively). For each correlation test, the Pearson correlation index is given and associated p-value in parenthesis.

| Fish group | |  | Eff_-1_ | | Eff_-2_ | | *L*_-1_ | | *H*_-1_ | | *L*_-2_ | | *H*_-2_ | | *L*_-3_ | | *H*_-3_ | |  |
| --- | --- | --- | --- | --- | --- | --- | --- | --- | --- | --- | --- | --- | --- | --- | --- | --- | --- | --- | --- |
| All-species | | Eff | 0.29 | | -0.17 | | -0.32 | | 0.17 | | 0.18 | | -0.13 | | 0.27 | | -0.43 | |  |
|  |  |  | (0.3553) | | (0.5977) | | (0.3175) | | (0.6038) | | (0.5751) | | (0.6769) | | (0.3969) | | (0.1660) | |  |
|  | Piscivore | Eff | | 0.19 | | -0.02 | | -0.29 | | 0.12 | | 0.30 | | -0.16 | | 0.22 | | -0.36 | |
|  |  |  | | (0.5606) | | (0.9580) | | (0.3604) | | (0.7023) | | (0.3430) | | (0.6135) | | (0.4952) | | (0.2478) | |
|  | Omnivore | Eff | | 0.13 | | -0.06 | | -0.44 | | 0.24 | | 0.34 | | -0.35 | | 0.35 | | -0.36 | |
|  |  |  | | (0.6788) | | (0.8596) | | (0.1494) | | (0.4514) | | (0.2827) | | (0.2705) | | (0.2611) | | (0.2451) | |
|  | Herbivore | Eff | | 0.35 | | -0.17 | | -0.65 | | 0.19 | | 0.21 | | -0.37 | | 0.29 | | -0.17 | |
|  |  |  | | (0.2589) | | (0.5951) | | (0.0210) | | (0.5497) | | (0.5104) | | (0.2356) | | (0.3684) | | (0.5973) | |
|  | Detritivore | Eff | | 0.32 | | -0.31 | | -0.45 | | 0.47 | | -0.09 | | -0.22 | | 0.33 | | -0.49 | |
|  |  |  | | (0.3171) | | (0.3183) | | (0.1473) | | (0.1271) | | (0.7695) | | (0.4889) | | (0.2866) | | (0.1075) | |

**Appendix table 5.** Mean annual yield and effort values and respective standard deviation (std dev) and coefficients of variation (C.V.).

| Guild | Yield (kg) | | | Effort (fisher*day) | | |  |
| --- | --- | --- | --- | --- | --- | --- | --- |
|  | Mean | Std dev | C.V. (%) | Mean | Std dev | C.V. (%) | |
| All-species | 2,542,586 | 709,223 | 28 | 123,361 | 31,955 | 26 | |
| Piscivores | 831,095 | 301,404 | 36 | 85,272 | 24,779 | 29 | |
| Omnivores | 196,455 | 49,366 | 25 | 36,949 | 9,680 | 26 | |
| Herbivores | 106,627 | 39,576 | 37 | 26,133 | 9,068 | 35 | |
| Detritivores | 475,479 | 195,554 | 41 | 31,861 | 12,641 | 40 | |

**Appendix table 6.** Mean flood pulse values and respective standard deviation (std dev) and coefficients of variation (C.V.).

| Flood-pulse index | Mean (cm*days) | Std dev | C.V. (%) |
| --- | --- | --- | --- |
| *H_-1_* | 57,467 | 16,408 | 29 |
| *H_-2_* | 58,022 | 16,393 | 28 |
| *H_-3_* | 57,427 | 16,339 | 28 |
| *L_-1_* | 18,783 | 8,841 | 47 |
| *L_-2_* | 19,941 | 9,309 | 47 |
| *L_-3_* | 20,357 | 9,108 | 45 |

**Appendix table 7.** Semi-partial R^2^ of explanatory variables of best approximating models.

| Fish groups | Semi-partial R^2^ of explanatory variables | | | |
| --- | --- | --- | --- | --- |
| All-species | Effort (89%) | *L_-2_* (9%) |  |  |
| Piscivore | Effort (92%) | *L_-2_* (5%) |  |  |
|  | Effort (88%) |  |  |  |
| Omnivore | Effort (48%) | *H_-2_* (13%) |  |  |
|  | Effort (58%) | *L_-3_* (9%) | *H*_-3_ (14%) |  |
| Herbivore | Effort (40%) | *L_-2_* (25%) | *H*_-2_ (13%) |  |
|  | Effort (53%) |  |  |  |
|  | Effort (65%) | *L_-2_* (15%) |  |  |
|  | Effort (35%) | *L_-3_* (13%) |  |  |
|  | Effort (42%) | *H_-1_* (12%) |  |  |
| Detritivore | Effort (82%) |  |  |  |
